# 3-Hydroxyanthranilic acid Administration Did Not Prevent Age Related Bone Loss

**DOI:** 10.1093/geroni/igab046.2542

**Published:** 2021-12-17

**Authors:** Carlos Isales, Ke-Hong Ding, Wendy Bollag, Meghan McGee-Lawrence, William Hill, Xing-ming Shi, Sadanand Fulzele, Mark Hamrick

**Affiliations:** 1 Medical College of Georgia, Augusta, Georgia, United States; 2 Augusta University, augusta, Georgia, United States; 3 Augusta University, Augusta, Georgia, United States; 4 Medical College of Georgia, Augusta University, Augusta, Georgia, United States; 5 Medical University of South Carolina, Charleston, South Carolina, United States

## Abstract

Aging is associated with accumulation of various tryptophan degradation products that may having either bone anabolic or catabolic effects. In epidemiologic studies, elevated levels of 3-hydroxyanthranilic acid (3-HAA) are associated with a higher bone mineral density (BMD). We have previously shown that the C57BL/6 mouse loses bone mass with age. Thus, we hypothesized that administering 3-HAA via a daily intraperitoneal (IP) injection would result in preserved or increased BMD. In an IACUC-approved protocol, we injected 26-month-old C57BL/6 mice with either a low dose (0.5 mg) or high dose (5 mg) of 3-HAA IP five days a week for eight weeks. At the end of this time mice were sacrificed and body composition and bone mineral density measured by DigiMus. BMD was significantly lower in the high dose 3-HAA group: 0.0570 + 0.004 vs 0.0473 + 0.006 vs 0.0432 + 0.0075 gm/cm2, (means+SD, Control vs 0.5 mg 3HAA vs 5 mg 3HAA, p=0.004, 0 vs 5.0 mg, n=6-9/group). 3-HAA had no significant impact on body composition (lean body mass: 86.7 + 1.7% vs 86.2 + 2.7% vs 86.1 + 2.0%, Control vs 0.5 mg vs 5.0 mg 3-HAA, p=ns; and fat mass 12.6 + 2.0% vs 13.8 + 2.7% vs 13.9 + 2.0% vs 0.2%, Control vs 0.5 vs 5 mg 3-HAA, p=ns). Thus, 3-HAA did not prevent bone loss in older mice but instead significantly worsened bone loss. 3-HAA is known to have both pro- and anti- oxidant effects depending on the environment. These data would suggest that at the higher concentrations 3-HAA is acting predominantly as a pro-oxidant molecule accelerating age-related bone loss.

